# The Hippo Pathway in Innate Anti-microbial Immunity and Anti-tumor Immunity

**DOI:** 10.3389/fimmu.2020.01473

**Published:** 2020-08-04

**Authors:** Qian Zhang, Ruyuan Zhou, Pinglong Xu

**Affiliations:** ^1^MOE Laboratory of Biosystems Homeostasis & Protection and Innovation Center for Cell Signaling Network, Life Sciences Institute, Zhejiang University, Hangzhou, China; ^2^Zhejiang Provincial Key Laboratory of Pancreatic Disease, Department of Hepatobiliary and Pancreatic Surgery, The First Affiliated Hospital, Zhejiang University School of Medicine, Hangzhou, China

**Keywords:** Hippo-YAP signaling, innate immunity, anti-tumor immunity, anti-microbial, nucleic acid sensing, antiviral immunity, interferon, cGAS-STING

## Abstract

The Hippo pathway responds to diverse environmental cues and plays key roles in cell fate determination, tissue homeostasis, and organ regeneration. Aberrant Hippo signaling, on the other hand, has frequently been implicated in diversified pathologies such as cancer and immune dysfunction. Here, we summarize the recent but rapid progress in understanding the involvement of the Hippo pathway in innate immunity, with a special focus on the intrinsic mechanisms and mutual interactions between Hippo-YAP signaling and the innate immune response and its physiological impacts on anti-microbial immunity and anti-tumor immunity. Moving forward, we believe that systematic investigations under the physiological setting are needed to draw a clearer picture of the actions of Hippo in innate immunity.

## Introduction

The Hippo pathway responds to diverse extracellular cues and plays key roles in tissue homeostasis, organ regeneration, and tumorigenesis. Originally discovered in *Drosophila*, the Hippo pathway is highly conserved in evolution (1–4). A kinase cascade by four tumor suppressors constitutes the core of the Hippo pathway, comprising two signaling complexes—the Hpo-Sav (MST-SAV in mammals) and the Wts-Mat (LATS-MOB in mammals), which govern the cellular localization, activity, and fate of signaling effectors YAP and TAZ (1, 5–8). Transcription coactivators YAP and TAZ therefore serve as downstream effectors in response to unfavorable growth conditions such as those derived from mechanical signals, cell adhesion, GPCR ligands, and cellular stresses and instructed by upstream kinases including MST, MAP4Ks, TAO, and AMPK (9–19). Activated LATS1/2 kinases directly phosphorylate five conserved serine residues on YAP (20), which drives the binding of YAP to 14-3-3 proteins for sequestration and cytoplasmic retention (21, 22) as well as the ubiquitination and proteasomal degradation (20, 23, 24). Otherwise, YAP/TAZ are localized in the nucleus to form transcription complex and activate the TEAD family transcription factors (25, 26), thereby transcribing target genes to promote cell proliferation, survival, and migration (27).

In *Drosophila*, mutations in major kinases of the Hippo pathway (Hpo/Wts) or their upstream regulators (Ex, Mer, Kibra, Ft, etc.) lead to overgrowth of eyes, wings, and other organs, mainly due to a sustained Yki activation that induces excessive cell proliferation (23, 28). Correspondingly, a sustained activation of YAP in mouse livers leads to cell transformation and tumor formation (22), while knocking out of YAP in mouse tissues results in various abnormalities in heart, skin, and kidney (29–32). YAP and TAZ are also highly active in stem cells and ancestral cells of various tissues and play important roles in the maintenance of stemness (33). TAZ is also involved in maintaining the self-renewal of breast cancer stem cells (34), while YAP causes genomic instability in medulloblastoma (35). Taken together, these observations indicate that the activities of YAP/TAZ resulting from Hippo signaling are key factors in cell fate determination and tissue homeostasis.

## Signaling Regulation and Crosstalks of the Hippo Pathway

**Figure 1:**
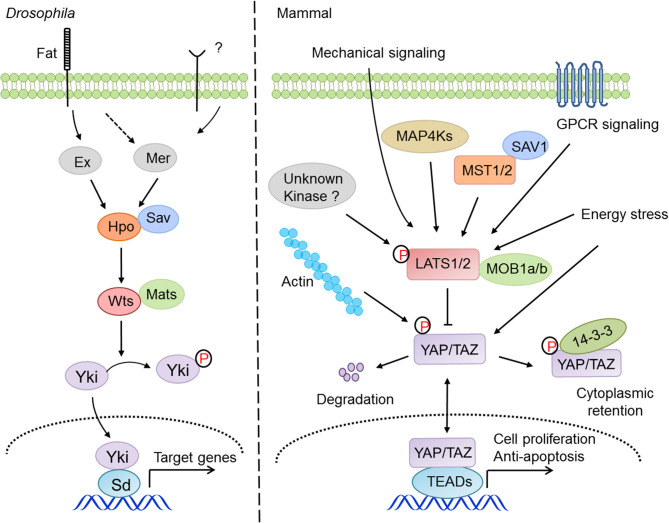
The signaling mechanism and regulation of the Hippo pathway in drosophila and mammals. The Hippo pathway answers to a variety of intracellular and extracellular cues and regulates cell proliferation, survival, and migration via the signaling cascade of MST1/2-LATS1/2-YAP/TAZ-TEADs.

Since its discovery, the physiological and pathological aspects of the Hippo pathway have been steadily established, particularly in development, homeostasis, and regeneration of organs, including liver, heart, intestine, brain and central nervous system (CNS), lung, and kidney (36), and in diseases of cancer, immune disorder, and cardiovascular dysfunction (27). The regulation of Hippo-YAP signaling appears to be very complicated (Figure 1), and there are crosstalks with the Notch pathway (37), the TGF-β pathway (38), the WNT pathway (39), G protein-coupled receptor (GPCR) signaling (11, 40), and innate immune signaling (41, 42). Recently, two studies have revealed that the MAP4K kinase family, including MAP4K1/2/3/5 and MAP4K4/6/7, directly phosphorylate and activate LATS1/2 independent of MST1/2 (13, 14), indicating the existence of other kinases involved in LATS1/2 regulation under the distinct tissue and signaling niches. Moreover, YAP and TAZ are targeted by many other kinases such as AMPK (17, 18), CDK1 (20), JNK (43), HIPK (44), and the tyrosine kinases c-Abl (45) and the Src family (46–49), indicating that YAP and TAZ possess a variety of additional regulations independent of the Hippo pathway. One of the major research focuses regarding the Hippo pathway is on understanding how it integrates with cellular intrinsic factors and cooperates with the other signaling pathways to regulate a myriad of physiological and pathological processes. In this review, we are focusing our discussions of the Hippo-YAP pathway on a specific topic, innate immunity.

## The Function of the Hippo Pathway in Innate Immune Cells

By regulating the transactivation of TEAD family transcription factors, Hippo-YAP signaling exerts critical roles in cell proliferation, apoptosis, migration, and pluripotency (50). Recently, accumulating evidence has suggested that the Hippo pathway is considerably involved in regulation of the differentiation, metabolism, and functions of innate immune cells (51–56). Macrophages and dendritic cells (DCs) are pivotal types of innate immune cells connecting the innate and adaptive immunity (57–59). Under physiological conditions, macrophages are polarized into M1 type, which mainly engages in pro-inflammatory and anti-tumor responses, or differentiated into the M2 type, which is mainly responsible for anti-inflammatory and pro-tumorigenic signals (60, 61). Activated YAP/TAZ in tumor cells and hepatocyte appears to be an effective attractant to drive the tissue infiltration of macrophages in the contexts of the tumor immune-environment and organ fibrosis, although the expression levels of YAP/TAZ in macrophages are still a controversial topic (62). For example, the activation of YAP in hepatocellular carcinoma underlies the M2 macrophage recruitment by tumor-initiating cells (TICs) (63) or promotes the migration and infiltration of M1-like macrophages (53), while the genetic deletion of Mst1/2 in hepatocytes upregulates MCP1 expression and causes a massive infiltration of macrophages with mixed M1 and M2 phenotypes, which promotes the development of HCC (64). A recent report also showed that YAP in macrophages impaired the IL-4/IL-13-induced M2 macrophage polarization but meanwhile promoted the LPS/ IFN-γ-triggered activation of M1 macrophage, which produced excessive IL-6 to aggravate intestinal bowel disease (IBD) (65). In another report, CYR61, but not CCL2/CSF1 (63), functioned as the downstream factor of YAP/TAZ in hepatocytes to attract liver macrophage infiltration, leading to liver inflammation and fibrosis (66). These intriguing observations may reflect context- and cellular type-dependency of the Hippo pathway in the regulation of macrophage polarization and tissue infiltration.

On the other hand, less is known about the role of the Hippo pathway in DCs, another major group of antigen-presenting cells (APCs) that present antigens to CD8^+^ T cells and activate cytotoxic T cells to obliterate virus, bacteria, and tumor cells (67–69). Recently, the Chi group found that the DC-specific deletion of MST1/2, but not LATS kinases or YAP/TAZ, leads to selective disruption of the homeostasis and function of CD8α^+^ T cells (51). They revealed that CD8α^+^ DCs exhibited a particularly robust oxidative metabolism that critically relies on MST1/2 signaling to maintain both the bioenergetic activities and mitochondrial dynamics. As a result, MST1/2-deficient CD8α^+^ DCs were impeded in the presentation of extracellular proteins and cognate peptides to prime CD8^+^ T cells (51). This report unveils the intriguing interaction between immune signaling and metabolic reprogramming that underlies the unique function of a subset of DCs. In addition, Torres-Bacete and colleagues revealed that depending on CCR7-RhoA signaling, MST1 selectively regulated the cyto-architecture, endocytosis, and migratory speed of mature dendritic cells (mDCs) (55). Taken together, these studies suggest that the Hippo pathway may participate in the homeostasis and function of DCs via distinct mechanisms. The functions of the Hippo pathway in innate immune cells are usually independent of LATS kinases and YAP/TAZ effectors, due to the general deficiency of YAP and TAZ expression in these cells.

## The Hippo Pathway in Regulation of Innate Immune Signaling

Innate immunity, which presents in both immune cells and non-immune cells, functions as the first defense line of defense against pathogen invasion. The host recognizes the pathogen-associated molecular patterns (PAMPs) of pathogens, and this triggers immune responses via numerous pattern recognition receptors (PRRs) (70), including membrane-anchored Toll-like receptors (TLRs) and C-type lectin receptors (CLRs) (71, 72), which are distributed mainly in immune cells, and cytosolic receptors such as RIG-I-like receptors (RLRs) (73–76), Cyclic GMP-AMP synthase (cGAS) (77–80), NOD-like receptors (81, 82), and AIM2-like receptors (83, 84), which have widespread expression. The Hippo pathway appears considerably involved in the regulation of innate immunity during pathogen infection, which is largely distinct from its canonical roles in organ growth control and tissue homeostasis maintenance. Our group and others have revealed that YAP and TAZ functioned as potent suppressors to compromise the production and signaling of type I interferons (IFN-Is) (41, 85, 86) and the activation of NF-κB (42) and that they served as positive regulators for differentiation of the Treg lymphocytes (87). For example, Zhang et al. revealed that YAP and TAZ acted as potent suppressors of TBK1, the central kinase in innate nucleic acid-sensing signaling (33); Deng et al. revealed that YAP/TAZ attenuated NF-κB signaling by directly inhibiting IKKα/β activation in an osteoarthritis murine model (88); Ni et al. found that YAP was highly expressed in Treg cells to amplify TGFβ-SMAD activation, which strengthened Foxp3 expression and Treg functions (87). These observations are supported by the greater severity of inflammatory phenotypes and the elevated anti-tumor immunity in mice with YAP and/or TAZ deficiency (87–92). Studies from several independent groups also suggested that YAP activation facilitated the expression of SOCS3 and suppressed the JAK-STAT inflammatory cascade in astrocytes (89), and during vascular inflammation, prevented NF-κB signaling by associating with TRAF6 and facilitating its degradation (90). In the case of myocardial fibrosis, YAP in epicardium promoted the recruitment of suppressive immune cells Tregs and thus suppressed the post-infarct inflammatory response and myocardial fibrosis (91). These observations under various physiological or pathological conditions thus suggest that YAP and TAZ function as suppressors of innate immune signaling and inflammatory responses.

On the other hand, the Guan group reported that deletion of kinases LATS1/2, which resulted in YAP/TAZ activation, induced the secretion of nucleic acid-rich extracellular vesicles (EVs) to promote IFN-I production, dendritic cell maturation, and CD8^+^ T-cell expansion and eventually contributed to anti-tumor immunity (93). YAP was also shown to promote NF-κB signaling by suppressing USP31, a negative regulator of NF-κB signaling, which resulted in the acceleration of sarcomagenesis (94). In endothelial cells, the activity of YAP/TAZ was suppressed by atheroprotective unidirectional shear stress, which resulted in downregulated expression of pro-inflammatory genes and decreased monocyte infiltration (95). Therefore, the roles of YAP/TAZ in innate immune signaling and inflammation appear to be highly context-dependent.

## The Hippo Pathway in Innate Anti-Bacterial Immunity

Organisms from drosophila to mammals are widely armed with TLR-mediated anti-microbial responses. The Pan group found that silencing of the Hippo pathway or activation of Yorkie in drosophila fat bodies led to an increase in cactus expression, which suppressed NF-κB signaling and thereby decreased the production of anti-microbial peptides, eventually compromising the resistance of host to gram-positive bacteria (42). Upon bacterial infection, the Hippo pathway in Drosophila enterocytes was coupled with the TGF-β and Src-MAPK pathways to upregulate the transcription of upd3, which contributed to intestinal stem cell-dependent tissue repair (96). In contrast, LegK7, the effector kinase of *L. pneumophila*, could mimic the host MST1 kinase to trigger the degradation of YAP/TAZ, which interfered with PPARγ activity and altered the transcriptional profile to impair macrophage anti-bacterial immunity (97). Therefore, the precise mechanisms and physiological roles of Hippo-YAP signaling in innate anti-bacterial immunity may await further investigation.

**Figure 2:**
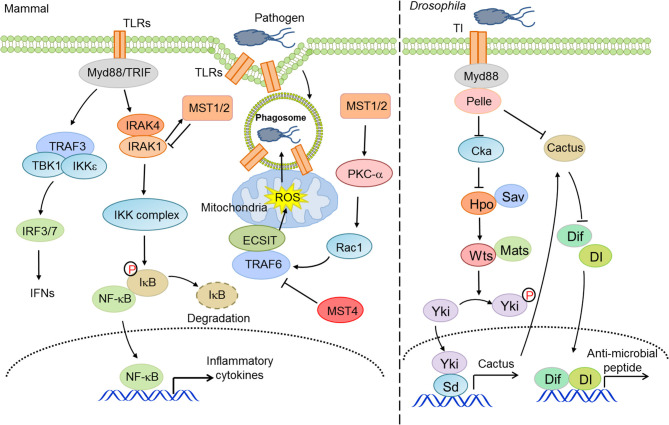
The Hippo pathway in innate anti-bacterial immunity. Hippo-YAP signaling is considerably involved in innate anti-bacterial immunity at multiple layers to address the activity of TLRs and NF-κB and ROS production.

On the other hand, several studies have unveiled that other core components of the Hippo pathway, independent of the canonical effectors YAP/TAZ, engage in the regulation of innate anti-bacterial immunity. For instance, MST1/2 kinases promoted mitochondria trafficking and reactive oxygen species (ROS) production in phagocytes (98), which facilitated anti-bacterial responses and bactericidal activity (99). Mechanistically, MST1/2 facilitated the formation of TLR-triggered TRAF6-ECSIT complex, which recruited mitochondria to phagosomes and accelerated the production of mitochondrial ROS (98). Intriguingly, MST1 disrupted the secretion of TLR4/9-triggered inflammatory cytokines to avoid chronic inflammation in hepatocellular carcinomas (HCCs) via priming the degradation of IRAK1 in macrophages (100). MST4, a homologous kinase of MST1/2, also restricted inflammatory responses via the phosphorylation of TRAF6 (101). Taken together, these studies suggest an important but complicated role for the Hippo pathway, canonical or non-canonical, in innate anti-bacterial immunity (Figure 2).

## The Hippo-YAP Pathway in Innate Antiviral Immunity

**Figure 3:**
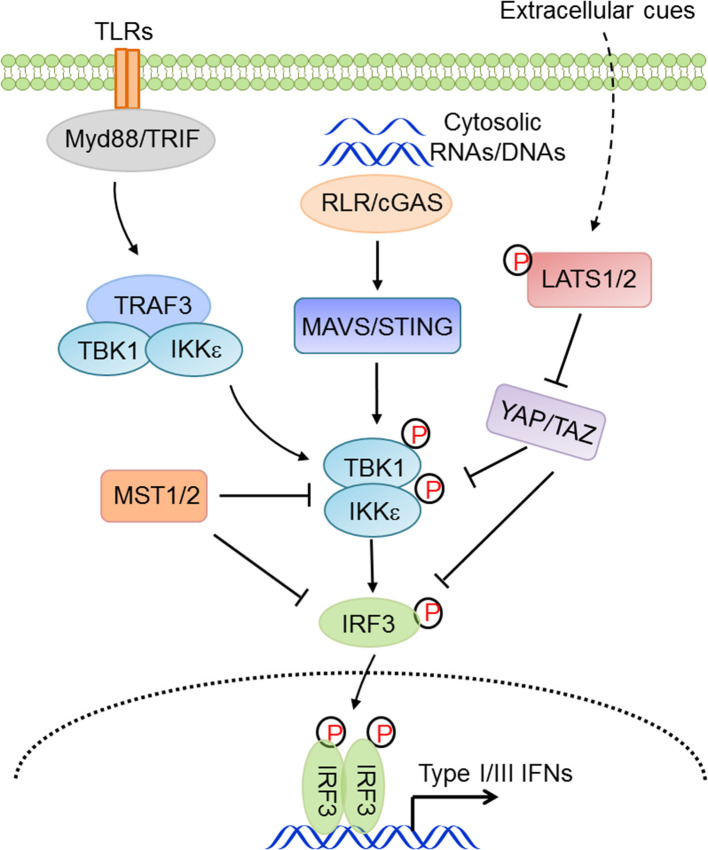
The Hippo pathway in innate antiviral immunity. Hippo-YAP signaling connects the extracellular cues to innate antiviral responses via complex regulations at multiple levels and in distinct physiological contexts.

In vertebrates, cytoplasmic nucleic acid sensing, which monitors both foreign and aberrant nucleic acids in the cytosol, is an essential component of innate antiviral immunity. RIG-I-like receptors (RLRs, including RIG-I and MDA5) surveil for heterogonous or aberrant RNA molecules (73–76), while abnormal nuclear or mitochondrial DNA molecules are primarily sensed by cGAS (77–80), which initiates cGAS-STING signaling, or by Aim2, which initiates the inflammasomal program. Facilitated by mitochondrial or endoplasmic reticulum-localized adaptor proteins MAVS/VISA/IPS-1/Cardif (102–104) or STING/MITA/ERIS (105, 106), nucleic acid sensing activates TBK1 and IKKε, which are responsible for phosphorylation and mobilization of IRF3. Activated IRF3 is then dimerized and translocated into the nucleus where it functions as a transcriptional factor by synergizing with NF-κB to transcribe type I IFNs and ISGs, eventually establishing an appropriate immune state and modulating the adaptive immunity (107, 108). Our group found that YAP and TAZ functioned as intrinsic inhibitors of TBK1, being directly associated with TBK1 and preventing the K63-linked ubiquitination and activation of TBK1 (41). In contrast, cellular conditions favoring YAP/TAZ inactivation and degradation relieved this YAP/TAZ-mediated TBK1 suppression and thus augmented the antiviral immunity (41). Zhou and colleagues also showed a few months later that YAP in macrophages negatively regulated the antiviral response via interfering with IRF3 dimerization and nuclear translocation (85). Other groups also revealed that YAP/TAZ attenuated NF-κB signaling by directly inhibiting IKKα/β activation in an osteoarthritis murine model (88) and by associating with TRAF6 and facilitating TRAF6 degradation in vascular inflammation (90). Recently, the Yu group reported that an isoform of TAZ impeded JAK-STAT signaling to dampen the type I IFNs pathway (86). In addition, our group found that, independent of LATS kinases and YAP/TAZ, MST1 directly associated with and phosphorylated IRF3 to attenuate its dimerization and promoter binding (109), and our and other groups further revealed that MST1 was also suppressive of TBK1 activation (109, 110). During HIV-1 infection, the CLRs DC-SIGN recognized the abortive HIV-1 RNA and triggered Raf-1-dependent MST1 activation, which facilitated the phosphorylation and activation of mitotic kinase PLK1 to restrain TBK1 and thus compromise MAVS antiviral signaling (110). Given the overall deficiency of YAP/TAZ expression in many types of immune cells, the observations with regard to the similarity but not the distinction of YAP/TAZ and MST1 in innate immunity are rather intriguing. Taken together, these observations suggest a considerable involvement of the Hippo pathway in innate antiviral immunity and the negative effects of YAP/TAZ and MST1 in antiviral immunity, depending on the distinct physiological contexts (Figure 3).

## Emerging Roles of Hippo-YAP Signaling in Modulation of Innate Anti-Tumor Immunity

**Figure 4:**
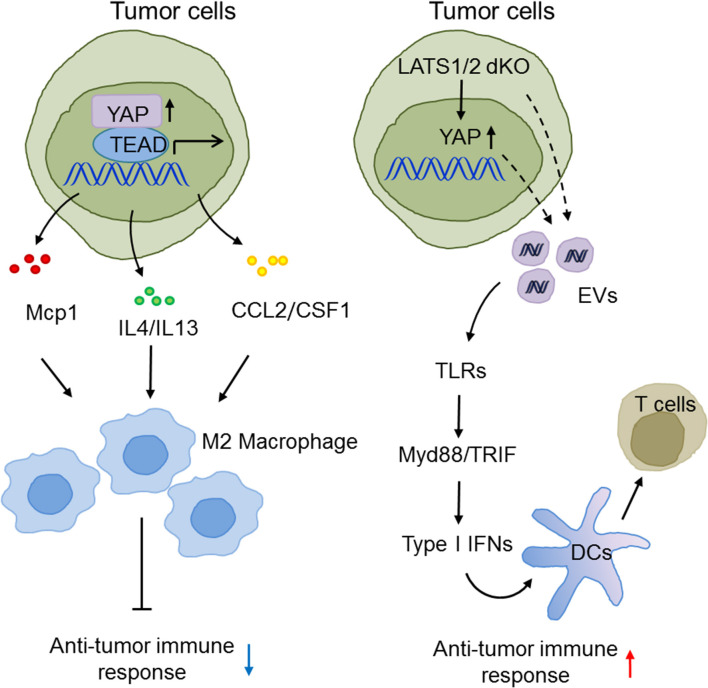
The emerging roles of Hippo-YAP signaling in the modulation of innate anti-tumor immunity. The Hippo pathway maneuvers anti-tumor immune responses and tumorigenesis via regulating the production of type I IFNs and the infiltration, polarization, and maturation of innate immune cells.

Type I IFNs bridge innate immunity and adaptive immunity and play a pivotal role in anti-tumor immunity (111–113). Given that there are numerous lines of evidence for the participation of the Hippo pathway in innate immunity, it is no surprise that Hippo-YAP signaling also effects anti-tumor immunity. For example, the Guan group suggested an unexpected function of the Hippo pathway in suppression of anti-tumor immunity in three murine models. Tumor cells with LATS1/2 deficiency secreted nucleic acid-rich EVs to facilitate TLRs-Myd88/TRIF signaling and type I IFN production, which accelerated DC maturation, CD8^+^ T lymphocyte expansion, and tumor growth arrest (93). In contrast, the Zhao group found that in liver tumor-initiating cells (TICs), YAP recruited M2 macrophages to suppress immune clearance, thus promoting tumorigenesis (63). In human HCC, the Yang group also reported that YAP activation induced the polarization of M1/M2 macrophages via Mcp1, thus contributing to massive macrophage infiltration and HCC progression (64). The Dey group reported that hyperactivated TAZ, but not YAP, accelerated liver inflammation and tumor development in a TEAD-dependent manner to induce myeloid cell infiltration and pro-inflammatory cytokine secretion (114). These somewhat diverse observations may reflect the fact that the complex tumor microenvironment and cancer cell types precisely determine the exact role of the Hippo-YAP signaling pathways in innate anti-tumor immunity. Future investigations are warranted to define these elaborate situations (Figure 4).

## Concluding Remarks

Contrary to the classic functions of Hippo-YAP signaling in cell fate determination, tissue homeostasis, and organ development, accumulating data in recent years has clearly pointed to the critical roles of the Hippo-YAP pathway in innate immune regulation. In this review, we have summarized the intriguing but complex integrations of Hippo-YAP signaling in the functioning of innate immune cells and signaling with specific focuses on the innate anti-microbial immunity and anti-tumor immunity. An intrinsic part of the nature of the Hippo pathway is its very complicated interactions with innate immune signaling at multiple levels and independent or dependent of signaling effectors YAP/TAZ. The entirely distinct programs for the expression of these core signaling players in innate immune cells, particularly YAP and TAZ, appear to justify this requirement.

In conclusion, Hippo signaling through the kinase cascade to YAP/TAZ is a conceptually straightforward pathway. However, we have observed the amazing versatility and context-dependence of the Hippo pathway responses in innate immunity. Moving forward, we believe that systematic investigations under physiological settings are particularly needed to decipher the diversified functions and mechanisms of the Hippo pathway, which, ultimately, will guide us to maneuver this critical pathway to benefit our health.

## Author Contributions

QZ and PX reviewed the literature and wrote the manuscript. RZ contributed with data analyses and discussions. All authors contributed to the article and approved the submitted version.

## Conflict of Interest

The authors declare that the research was conducted in the absence of any commercial or financial relationships that could be construed as a potential conflict of interest.
